# Maternal outcomes and risk factors for COVID-19 severity among pregnant women

**DOI:** 10.1038/s41598-021-92357-y

**Published:** 2021-07-06

**Authors:** Manon Vouga, Guillaume Favre, Oscar Martinez-Perez, Leo Pomar, Laura Forcen Acebal, Alejandra Abascal-Saiz, Maria Rosa Vila Hernandez, Najeh Hcini, Véronique Lambert, Gabriel Carles, Joanna Sichitiu, Laurent Salomon, Julien Stirnemann, Yves Ville, Begoña Martinez de Tejada, Anna Goncé, Ameth Hawkins-Villarreal, Karen Castillo, Eduard Gratacos Solsona, Lucas Trigo, Brian Cleary, Michael Geary, Helena Bartels, Feras Al-Kharouf, Fergal Malone, Mary Higgins, Niamh Keating, Susan Knowles, Christophe Poncelet, Carolina Carvalho Ribeiro-do-Valle, Fernanda Surita, Amanda Dantas-Silva, Carolina Borrelli, Adriana Gomes Luz, Javiera Fuenzalida, Jorge Carvajal, Manuel Guerra Canales, Olivia Hernandez, Olga Grechukhina, Albert I. Ko, Uma Reddy, Rita Figueiredo, Marina Moucho, Pedro Viana Pinto, Carmen De Luca, Marco De Santis, Diogo Ayres de Campos, Inês Martins, Charles Garabedian, Damien Subtil, Betania Bohrer, Maria Lucia Da Rocha Oppermann, Maria Celeste Osorio Wender, Lavinia Schuler-Faccini, Maria Teresa Vieira Sanseverino, Camila Giugliani, Luciana Friedrich, Mariana Horn Scherer, Nicolas Mottet, Guillaume Ducarme, Helene Pelerin, Chloe Moreau, Bénédicte Breton, Thibaud Quibel, Patrick Rozenberg, Eric Giannoni, Cristina Granado, Cécile Monod, Doris Mueller, Irene Hoesli, Dirk Bassler, Sandra Heldstab, Nicole Ochsenbein Kölble, Loïc Sentilhes, Melissa Charvet, Jan Deprest, Jute Richter, Lennart Van der Veeken, Béatrice Eggel-Hort, Gaetan Plantefeve, Mohamed Derouich, Albaro José Nieto Calvache, Maria Camila Lopez-Giron, Juan Manuel Burgos-Luna, Maria Fernanda Escobar-Vidarte, Kurt Hecher, Ann-Christin Tallarek, Eran Hadar, Karina Krajden Haratz, Uri Amikam, Gustavo Malinger, Ron Maymon, Yariv Yogev, Leonhard Schäffer, Arnaud Toussaint, Marie-Claude Rossier, Renato Augusto Moreira De Sa, Claudia Grawe, Karoline Aebi-Popp, Anda-Petronela Radan, Luigi Raio, Daniel Surbek, Paul Böckenhoff, Brigitte Strizek, Martin Kaufmann, Andrea Bloch, Michel Boulvain, Silke Johann, Sandra Andrea Heldstab, Monya Todesco Bernasconi, Gaston Grant, Anis Feki, Anne-Claude Muller Brochut, Marylene Giral, Lucie Sedille, Andrea Papadia, Romina Capoccia Brugger, Brigitte Weber, Tina Fischer, Christian Kahlert, Karin Nielsen Saines, Mary Cambou, Panagiotis Kanellos, Xiang Chen, Mingzhu Yin, Annina Haessig, Sandrine Ackermann, David Baud, Alice Panchaud

**Affiliations:** 1 Materno-fetal and Obstetrics Research Unit, Department “Femme-Mère-Enfant”, University Hospital, Lausanne, Switzerland; 2 Obstetricia y Ginecologia, Puerta de Hierro University Hospital, Madrid, Spain; 3 Obstetrics and Gynaecology Department, 12 de Octubre University Hospital, Madrid, Spain; 4 La Paz Universitary Hospital, Madrid, Spain; 5 Hospital Santa Caterina, Gerona, Spain; 6 Department of Obstetrics and Gynaecology, CHOG, Saint-Laurent du Maroni, France; 7 Obstétrique et de Médecine fœtale, Hopital Necker-Enfants Malades, Paris, France; 8 Obstetrics Division, Department of Pediatrics Gynecology and Obstetrics, University Hospitals of Geneva, Geneva, Switzerland; 9 BCNatal Maternal-Fetal Medicine Service, Hospital Clínic, University of Barcelona, Barcelona, Spain; 10 Fetal Medicine Research Center, Hospital Clínic and Hospital Sant Joan de Déu, Barcelona, Spain; 11 Service of Pharmacy, The Rotunda Hospital, Dublin, Ireland; 12 Maternal-fetal Medicine, The Rotunda Hospital, Dublin, Ireland; 13 UCD Perinatal Research Centre, National Maternity Hospital, Dublin, Ireland; 14 Maternal Medicine, National Maternity Hospital, Dublin, Ireland; 15 Microbiology, National Maternity Hospital, Dublin, Ireland; 16 Obstetric and Gynecology Unit, Centre Hospitalier René Dubos, Cergy-Pontoise, France; 17 Department of Obstetrics & Gynecology, University of Campinas, Campinas, Brazil; 18 Maternal-Fetal Medicine, Department of Obstetrics, Escuela de Medicina, Pontificia Universidad Católica de Chile, Santiago, Chile; 19 Medicina Materno Fetal, Hospital San José, Santiago, Chile; 20 Hospital Felix Bulnes Cerda, Santiago, Chile; 21 Department of Obstetrics, Gynecology and Reproductive Sciences, Yale School of Medicine, New Haven, CT USA; 22 Department of Epidemiology of Microbial Diseases, Yale of School of Public Health, New Haven, CT USA; 23 Serviço ginecologia e obstetrícia, Centro Hospitalar e Universitário São João, Porto, Portugal; 24 Teratology Information Service, Fondazione Policlinico Universitario Agostino Gemelli IRCCS, Rome, Italy; 25 Medical School, Santa Maria University Hospital, Lisbon, Portugal; 26 Department of Obstetrics, Jeanne de Flandre University Hospital, Lille, France; 27 Pediatra e Neonatologista, Hospital de Clinicas de Porto Alegre, Universidade Federal do Rio Grande do Sul, Porto Alegre, Brazil; 28 Maternity Ward, Hospital de Clinicas de Porto Alegre, Universidade Federal do Rio Grande do Sul, Porto Alegre, Brazil; 29 Departamento de Genética, Hospital de Clinicas de Porto Alegre, Universidade Federal do Rio Grande do Sul, Porto Alegre, Brazil; 30 Hospital de Clinicas de Porto Alegre, Universidade Federal do Rio Grande do Sul, Porto Alegre, Brazil; 31 Department of Obstetrics and Gynecology, Université de Franche Comté, Besançon, France; 32 Department of Obstetrics and Gynecology, Centre Hospitalier Departemental de Vendée, La Roche sur Yon, France; 33 Clinical Research Department, Centre Hospitalier Départemental de Vendée, La Roche sur Yon, France; 34 Department of Obstetrics and Gynecology, Annecy Genevois Hospital, Annecy, France; 35 Department of Gynecology and Obstetrics, Intercommunal Hospital Centre of Poissy-Saint-Germain-en-Laye, Poissy, France; 36 Department of Obstetrics and Antenatal Care, University Hospital Basel, Basel, Switzerland; 37 Department of Neonatology, UniversitätsSpital Zürich, Zurich, Switzerland; 38 Department of Anthropology, University of Zurich, Zurich, Switzerland; 39 Clinic of Obstetrics, UniversitätsSpital Zürich, Zurich, Switzerland; 40 Department of Obstetrics and Gynecology, Bordeaux University Hospital, Bordeaux, France; 41 Department of Obstetrics and Gynecology, University Hospitals Leuven, Leuven, Belgium; 42 Department of Regeneration and Development, Katholieke Universiteit Leuven, Leuven, Belgium; 43 Obstetric and Gynecology Unit, Sion Hospital, Sion, Switzerland; 44 Service de Réanimation polyvalente et USC, Victor Dupouy Hospital, Argenteuil, France; 45 Obstetrics Unit, Victor Dupouy Hospital, Argenteuil, France; 46 Obstetrics and Gynecology Department, Fundacion Clinica Valle de Lili, Universitary Hospital, Cali, Colombia; 47 Department of Obstetrics and Fetal Medicine, University Medical Center Hamburg-Eppendorf, Hamburg, Germany; 48 Maternal-Fetal Medicine Unit, Rabin Medical Center, Tel-Aviv University, Tel Aviv, Israel; 49 Division of Ultrasound in Obstetrics and Gynecology, Lis Maternity Hospital, Tel Aviv, Israel; 50 Division of Ultrasound in ObGy, Tel Aviv Sourasky Medical Center, Tel Aviv, Israel; 51 Israeli Society of Obstetrics and Gynecology, Hasaf Harofe Medical Center, Tel Aviv University, Tel Aviv, Israel; 52 Obstetrics Cantonal Hospital of Baden, Affiliated Hospital of the University of Zurich, Baden, Switzerland; 53 Department of Gynecology and Obstetrics, Intercantonal Hospital of Broye, Payerne, Switzerland; 54 Obstetrics and Gynecology, Hospital Riviera Chablais, Rennaz, Switzerland; 55 Maternal-Fetal Unit, Federal Fluminense University, Rio de Janeiro, Brazil; 56 Department of Obstetrics, Gynecology Stadtspital Triemli Zürich, Zurich, Switzerland; 57 Department of Infectious Diseases, University Hospital Bern, Bern, Switzerland; 58 Department of Obstetrics and Gynecology, Inselspital, Bern, Switzerland; 59 Department of Obstetrics and Prenatal Medicine, University Hospital Bonn, Bonn, Germany; 60 Obstetric and Gynecology Unit, Spital Bülach, Bülach, Switzerland; 61 Obstetric and Gynecology Unit, Hopital du Jura, Delémont, Switzerland; 62 Pôle Department of Gynecology and Obstetrics, GHOL Hôpital de Nyon, Nyon, Switzerland; 63 Department of Obstetrics and Gynecology Spitalzentrum OBerwallis, Standort Visp, Visp, Switzerland; 64 Frauenklinik, Kantonsspital Aarau, Aarau, Switzerland; 65 Department of Obstetrics and Gynecology, HFR Fribourg Hospital, Fribourg, Switzerland; 66 GynEcho Medical Practice, Fribourg, Switzerland; 67 Department of Obstetrics and Gynecology, La Rochelle Hospital, La Rochelle, France; 68 Department of Obstetrics and Gynecology, Ente Ospedaliero Cantonale of Lugano, Lugano, Switzerland; 69 Department of Obstetrics and Gynecology Réseau Hospitalier Neuchâtelois, Neuchâtel, Switzerland; 70 Department of Obstetrics and Gynecology, Kantonsspital Obwalden (KSOW), Sarnen, Switzerland; 71 Frauenklinik, Kantonsspital Saint Gall, Saint Gall, Switzerland; 72 Infectious Diseases and Hospital Epidemiology, Kantonsspital Saint Gall, Saint Gall, Switzerland; 73 Division of Infectious Diseases, Department of Pediatrics, David Geffen UCLA School of Medicine, Los Angeles, CA USA; 74 Cardiac Surgery Department, David Geffen UCLA School of Medicine, Los Angeles, CA USA; 75 Department of Obstetrics and Gynecology, Kantonsspital Uri, Altdorf, Switzerland; 76 Dermatology Unit, Xiangya Hospital, Changsha, China; 77 Hunan Engineering Research Center of Gynecology and Obstetrics Disease, Xiangya Hospital, Changsha, China; 78 Department of Obstetrics and Gynecology, Zuger Kantonsspital, Zug, Switzerland; 79 Institute of Primary Health Care (BIHAM), University of Bern, Bern, Switzerland; 80 Service of Pharmacy, Lausanne University Hospital and University of Lausanne, Lausanne, Switzerland; 81 Materno-Fetal & Obstetrics Research Unit, Department of Obstetrics and Gynecology, Centre Hospitalier Universitaire Vaudois (CHUV), 1011 Lausanne, Switzerland

**Keywords:** Risk factors, Infection, Clinical microbiology, Reproductive signs and symptoms, Respiratory signs and symptoms, Viral infection

## Abstract

Pregnant women may be at higher risk of severe complications associated with the severe acute respiratory syndrome coronavirus 2 (SARS-CoV-2), which may lead to obstetrical complications. We performed a case control study comparing pregnant women with severe coronavirus disease 19 (cases) to pregnant women with a milder form (controls) enrolled in the COVI-Preg international registry cohort between March 24 and July 26, 2020. Risk factors for severity, obstetrical and immediate neonatal outcomes were assessed. A total of 926 pregnant women with a positive test for SARS-CoV-2 were included, among which 92 (9.9%) presented with severe COVID-19 disease. Risk factors for severe maternal outcomes were pulmonary comorbidities [aOR 4.3, 95% CI 1.9–9.5], hypertensive disorders [aOR 2.7, 95% CI 1.0–7.0] and diabetes [aOR2.2, 95% CI 1.1–4.5]. Pregnant women with severe maternal outcomes were at higher risk of caesarean section [70.7% (n = 53/75)], preterm delivery [62.7% (n = 32/51)] and newborns requiring admission to the neonatal intensive care unit [41.3% (n = 31/75)]. In this study, several risk factors for developing severe complications of SARS-CoV-2 infection among pregnant women were identified including pulmonary comorbidities, hypertensive disorders and diabetes. Obstetrical and neonatal outcomes appear to be influenced by the severity of maternal disease.

## Introduction

Altered immunity, reduced respiratory capacity, vascular and hemodynamic changes put pregnant women at higher risk of complications, while specific harm to the exposed fetus/newborn may be observed. Although, early reports from the SARS-CoV-2 epidemic^1^ suggested that the clinical course for infected pregnant women was similar to the general population, more recent data suggest a higher risk of severe outcomes in pregnant women compared to the general population at an equivalent age, with severe outcomes observed in 8 to 11%^2–6^. In the general population, preexisting health conditions, namely pulmonary pathologies, hypertension and diabetes have been associated with severe outcomes^7,8^. Information on the impact of these determinants on the maternal disease evolution and other risk factors specific to pregnancy is still fragmented, although evidence suggest that they might contribute to the severity of the disease^6,9^. Furthermore, fetal/newborn risks still need to be better assessed as vertical transmission of the virus and placental infection appears to be possible with newborns potentially demonstrating related symptoms^10–13^, while a significantly higher rate of preterm deliveries (25–30%) among women with Coronavirus disease 19 (COVID-19) has been reported^3,4^.

Information on specific risks among pregnant women are urgently needed to provide evidence-based guidelines for the management of this vulnerable population. To accomplish this, we developed an international web registry^14^ in March 2020, to promote a structured collection of data regarding pregnant women and their fetuses exposed to SARS-CoV-2. Using this dataset, we performed a case–control study to assess the risk of severe maternal outcomes and associated risk factors as well as a description of pregnancy/neonatal outcomes stratified for the severity of the disease among pregnant women with a confirmed SARS-CoV-2 infection.

## Materials and methods

### Study setting and population

The patients enrolled in this study are part of the COVI-Preg international registry investigating the consequences of SARS-Cov-2 infection during pregnancy^14^. All pregnant women tested for SARS-CoV-2 infection at any stage of gestation were eligible for inclusion in this multicenter study except those < 18 years of age as well as individuals declining to consent or not able to consent for themselves. Informed oral or written consent was obtained for all participants. Deidentified data were prospectively recorded by each center (Table S1) using the REDCap (Research Electronic Data Capture) electronic data capture tool^15,16^. Quality checks were performed as described in the Supplementary Materials. Using this dataset, we performed a case control study among pregnant women with a confirmed SARS-CoV-2 infection.

The study was approved by both the Swiss Ethical Board (CER-VD-2020-00548) and the local ethics boards at each participating center. The study was conducted from March 24th to July 26th, 2020. All methods were carried out in accordance with relevant guidelines and regulations in the manuscript.

### Inclusion criteria and SARS-CoV-2 status

Pregnant women were tested for SARS-CoV-2 either because of a suspected infection due to ongoing symptoms compatible with COVID-19 or an history of potential exposure or through routine systematic screening instituted during the pandemic in some hospitals depending on local capacities and guidelines. Maternal testing was performed using a nasopharyngeal RT-PCR for SARS-CoV-2 swab test. Pregnant women with a positive RT- PCR test result at any stage during pregnancy irrespective of clinical signs and symptoms were considered as having a confirmed infection and included in the present study. Pregnant women with a SARS-CoV-2 negative test and no other positive test result during the entire follow-up period were excluded.

### Case and control definition

Pregnant women with severe adverse outcomes, defined as any of the following: (1) the need for advanced oxygen support (i.e. high flow cannula, non-invasive ventilation through CPAP or mechanical ventilation), (2) admission to the intensive care unit (ICU) and (3) maternal death, were classified as cases. The control group included pregnant women with either mild adverse outcomes, defined as maternal hospitalization requiring oxygen supplementation, or no adverse outcomes*,* defined as outpatient management or hospitalization not requiring oxygen supplementation.

### Identification of risk factors for severe adverse maternal outcome

Pregnant women with severe adverse outcomes (cases) were compared to pregnant women with mild or no adverse outcomes (controls). The effect of maternal characteristics known to be risk factors^7,8,17^ for SARS-CoV-2 severe adverse outcomes in the general population were tested (i.e. maternal age > 35 years old, obesity defined as a BMI > 30, hypertensive disorders, pre-and gestational diabetes, preexisting pulmonary, cardiovascular, renal, or oncologic disease and immunosuppression), as well as pregnancy related risk factors such as nulliparity (dichotomized as yes/no), ethnicity (defined as Caucasian yes/no), multiple pregnancy, gestational age at infection (dichotomized as < or > 20 WG)^9^.

### Secondary outcomes: absolute risk (%) of obstetrical outcomes and neonatal outcomes

For completed pregnancies (i.e. pregnancy ending in either fetal loss > 14 WG or livebirth, obstetrical outcomes (pregnancy outcome, GA at delivery, mode of delivery) and neonatal outcomes (neonatal death, neonatal admission to the ICU (NICU), birthweight and rates of suspected perinatal SARS-CoV-2) were assessed. For multiple gestations (n = 26), the analysis considered the whole pregnancy. Fetal loss was defined as a spontaneous antepartum fetal death > 14 WG (i.e. late miscarriage (14–24 WG) and stillbirth (fetal demise > 24 WG). Suspected perinatal SARS-CoV-2 transmission was defined as a positive RT-PCR result performed at birth.

### Statistical analysis

We performed a multivariate analysis to estimate odds ratios (OR) with 95% CIs adjusting for risk factors of COVID-19 severity (i.e. maternal age, BMI, pre- and gestational hypertensive disorders (including pre-eclampsia), pre-and gestational diabetes, pre-existent pulmonary comorbidities, other pre-gestational comorbidities (cardiovascular, renal, oncological diseases and immunosuppression), and gestational risk factors of severe maternal outcomes (ethnicity, parity, pregnancy conditions (threatened preterm labor, placenta previa, placental malfunction and PPROM) and exposure after 20WG) and accounting for missing values as described in the supplementary material.

Statistical analyses were performed using Stata 14 (StataCorp. 2015. *Stata Statistical Software: Release 14*. College Station, TX: StataCorp LP). A *P* value less than 0.05 was considered as statistically significant.

## Results

**Figure 1:**
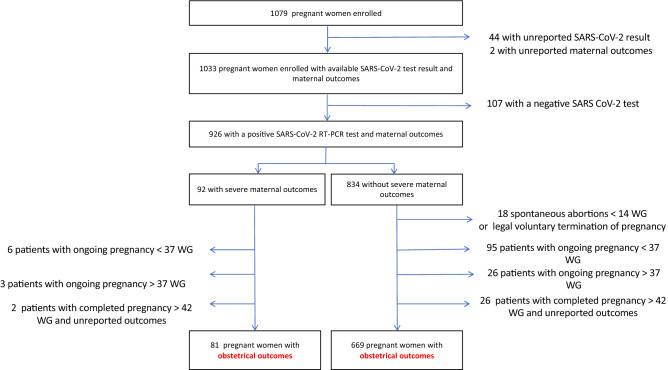
Flow chart. The COVI-Preg international registry was launched in March 2020. To date, 120 centers from 16 countries have contributed patients (supplementary Table 1). All pregnant women tested for SARS-CoV-2 infection at any stage of gestation were eligible for inclusion in this multicenter study except those < 18 years of age as well as individuals declining to consent or not able to consent for themselves. Deidentified data were prospectively recorded by each center using the REDCap (Research Electronic Data Capture) electronic data capture tool^15,16^. At inclusion (i.e. at the time of SARS-CoV-2 screening), the following data were recorded: socio-demographic characteristics, obstetrical history and information on SARS-CoV-2 exposure. Pregnancies were monitored as clinically indicated according to local protocols. After inclusion, the following data were collected: results of maternal testing (SARS-CoV-2 and/or other infectious pathogens), COVID-19 history, maternal, pregnancy and neonatal outcomes. Data were analyzed using Stata 14 (StataCorp. 2015. Stata Statistical Software: Release 14. College Station, TX: StataCorp LP). SARS-CoV-2, severe acute respiratory syndrome coronavirus 2WG, weeks ‘gestation.

Between March 24 and July 26, 2020, 1079 pregnant women tested for SARS-CoV-2 were enrolled in the registry among which 926 had a confirmed SARS-CoV-2 infection (Fig. 1). Socio-demographic characteristics are presented in Table 1. A third of the women were asymptomatic (31.9% n = 295/926), while cough (40.4%, n = 374/926), fever (32.4%, n = 300/926) and anosmia/ageusia (17.8%, n = 165/926) were the most reported symptoms. 9.9% (n = 92/926) experienced severe maternal outcomes, including 7.3% (n = 68/926) requiring advanced oxygen support and 4.0% (n = 37/926) requiring ICU admission; 6 maternal deaths were recorded (0.6%) (Table 2).

**Table 1:** Description of the population (sociodemographic characteristics).

Socio-demographic factors	Pregnant women with a confirmed SARS-CoV-2 infection (n = 926)
**Maternal age**	
Median—y.o. (IQR)	32 (28–36)
Age > 35 y.o.– no (%)	272 (29.4)
Unknown	5 (0.5)
**Ethnicity—no (%)**	
Caucasian	494 (53.4)
Hispanic or Latin-American	217 (23.4)
Afro-American	117 (12.6)
Asian or Pacific Islands	30 (3.2)
Other	44 (4.8)
Unknown	24 (2.6)
**Region of residence—no (%)**	
North America	27 (2.9)
South and Central America	249 (26.9)
Europe	490 (52.9)
Middle East	17 (1.8)
Central Asia	3 (0.3)
South East Asia	6 (0.6)
Africa	26 (2.8)
Unknown	108 (11.6)
**Previous pregnancies—no (%)**	
Nulliparous	346 (37.4)
Multiparous	568 (61.3)
Multiparous ≥ 3	102 (11.0)
Previous cesarean sections > 1	135 (14.6)
Unknown	12 (1.3)
**Previous adverse pregnancy outcomes—no (%)**	
Stillbirths	18 (1.9)
Unknown	163 (17.6)
**Maternal comorbidities**	
Any maternal comorbidities—no (%)	170 (18.4)
Pulmonary comorbidities	35 (3.8)
Cardiac comorbidities	14 (1.5)
Hypertension	19 (2.1)
Pregestational diabetes	12 (1.3)
Immunosuppression	4 (0.4)
Thyroid dysfunction	34 (3.7)
Oncologic comorbidities	9 (1.0)
Hematologic comorbidities	17 (1.8)
Auto-immune diseases	4 (0.4)
Other (neurological, urological, digestive, orthopedic)	85 (9.2)
Unknown	4 (0.4)
Maternal BMI	
Median (IQR)	26 (23–30)
BMI > 30—no (%)	208 (22.5)
BMI > 35—no (%)	81 (8.8)
Unknown—no (%)	122 (13.2)
Any drugs	63 (6.8)
Cigarettes	61 (6.6)
Alcohol	5 (0.5)
Unknown	17 (1.8)
**Current pregnancy—no (%)**	
Multiple pregnancy	24 (2.6)
Ongoing pregnancy conditions	
Any	114 (12.3)
Pre-eclampsia	10 (1.1)
Gestational diabetes	45 (4.9)
IUGR	7 (0.8)
Abnormal fetal doppler	1 (0.1)
Macrosomia	6 (0.7)
Threatening preterm labor	5 (0.5)
Placenta previa	2 (0.2)
PPROM	5 (0.5)
Other	46 (5.0)
Unknown	33 (3.6)
Fetal malformation	18 (1.9)
Risk of DS	
High risk > 1/1000	24 (2.6)
Unknown	341 (36.8)

SARS-CoV-2, severe acute respiratory syndrome coronavirus 2; y.o., years old; IQR, interquartile range; BMI, body mass index; PPROM, preterm premature rupture of the membranes; IUGR, intrauterine growth restriction; DS, Down syndrome; WG, weeks’ gestation.

**Table 2:** Description of the population (COVID-19 history).

COVID-19 history	Pregnant women with a confirmed SARS-CoV-2 infection (n = 926)
**Timing of exposure—no (%)**	
< 20 WG	89 (9.6)
Median GA at exposure WG (IQR)	12 (9–16)
> 20 WG	826 (89.2)
Median GA at exposure WG (IQR)	38 (34–40)
Unknown	11 (1.2)
**Clinical manifestation—no (%)**	
Asymptomatic	295 (31.9)
Fever	300 (32.4)
Cough	374 (40.4)
Dyspnea	146 (15.8)
Sore throat	83 (9.0)
Myalgia	148 (16.0)
Fatigue	191 (20.6)
Headache	121 (13.1)
Nausea/vomiting	48 (5.2)
Anosmia/ageusia	165 (17.8)
Other	81 (8.8)
**Maternal outcomes—no (%)**	
No adverse outcomes	828 (89.4)
Mild adverse outcomes	6 (0.6)
Severe adverse outcomes	92 (9.9)
Maternal deaths	6 (0.6)
Admission to ICU	37 (4.0)
Advanced oxygen support	68 (7.3)

First trimester was defined from 1 to 13 6/7 weeks’ gestation (WG), second trimester from 14 0/7 to 27 6/7 WG and third trimester from 28 WG. For symptomatic patients, trimester of exposure was defined as the gestational age (GA) at onset of symptoms. For asymptomatic patients, the trimester of exposure was defined as the GA at SARS-CoV-2 testing.

For symptomatic patients, the trimester of exposure was defined as the gestational age (GA) at onset of symptoms. For asymptomatic patients, the trimester of exposure was defined as the GA at SARS-CoV-2 testing.

IQR, interquartile range; ICU, Intensive Care Unit; WG, weeks’ gestation.

### Risk factors for severe maternal outcomes among positive pregnant women

In a univariate analysis pulmonary comorbidities [crude OR 3.9, 95% CI 1.6–8.9], hypertensive disorders [crude OR 3.5, 95% CI 1.2–9.1], diabetes [crude OR 2.6, 95% CI 1.2–5.3] and BMI > 30 [crude OR 1.7, 95% CI 1.1–2.9] were significantly associated with an increased risk of severe maternal outcomes (Table 3). In a multivariate analysis adjusting for risk factors of COVID-19 severity, gestational risk factors of severe maternal outcomes, and accounting for missing values through multiple imputation, pulmonary comorbidities [aOR 4.3, 95% CI 1.9–9.5], hypertensive disorders [aOR 2.7, 95% CI 1.0–7.0] and diabetes [2.2, 95% CI 1.1–4.5] remained significantly associated, while BMI > 30 did not retain significance [aOR 1.3, 95% CI 0.8–2.2]. When adjusting for COVID-19 risk factors only, similar results were obtained (Table 3). Common pregnancy related risk factors were not associated with severe maternal outcomes (i.e. nulliparity, ethnicity, multiple pregnancy, gestational age at infection).

**Table 3:** Risk factors for severe adverse maternal outcomes among pregnant women with a positive SARS-CoV-2 test.

Maternal outcomes	Pregnant women with a POSITIVE test result for SARS-CoV-2				OR^a^	95%CI	p value	aOR^b^	95%CI	p value	aOR^c^	95%CI	p value
	Severe adverse maternal outcomes n = 92		No/mild adverse maternal outcomes n = 834										
	n (%)	95% CI	n (%)	95% CI									
**Maternal age**													
Age > 35 y.o	28 (30.4)	21.3–40.9	244 (29.3)	26.2–32.5	1.0	0.6–1.7	0.9042	1.1	0.7–1.8	0.708	1.1	0.7–1.7	0.755
Unknown	0 (0.0)	n.a.	5 (0.6)	0.2–1.4									
**Ethnicity**													
Caucasian	41 (44.6)	34.2–55.3	453 (54.3)	50.9–57.7	0.7	0.4–1.1	0.0926	0.7	0.5–1.2	0.214			
Unknown	3 (3.3)	0.7–9.2	21 (2.5)	1.6–3.8									
**Previous pregnancies**													
Nulliparous—no (%)	29 (31.5)	22.2–42.0	317 (38.0)	34.7–41-4	0.8	0.5–1.2	0.2564	0.8	0.5–1.3	0.412			
Unknown	1 (1.1)	0.0–5.9	11 (1.3)	0.7–2.3									
**Maternal comorbidities gestational/pre-gestational**													
Pre-gestational comorbidities	19 (20.7)	12.9–35.7	123 (14.8)	12.4–17.3									
Pulmonary comorbidities	10 (10.9)	5.3–19.1	25 (3.0)	1.9–4.4	**3.9**	**1.6–8.9**	**0.0013**	**4.3**	**1.9–9.5**	**0.000**	**4.0**	**1.8–8.9**	**0.001**
Any other	6 (6.5)	2.6–13.7	40 (4.8)	0.7–6.5	1.4	0.5–3.4	0.4473	0.9	0.3–2.4	0.841	0.9	0.4–2.4	0.891
Cardiac comorbidities	3 (3.3)	0.7–9.2	11 (1.3)	0.7–2.3									
Renal diseases	2 (2.2)	0.3–7.6	4 (0.5)	0.1–1.2									
Immunosuppression	1 (1.1)	0.0–5.9	3 (0.4)	0.1–1.0									
Oncologic comorbidities	1 (1.1)	0.0–5.9	8 (1.0)	0.4–1.9									
Hematologic comorbidities	2 (2.2)	0.2–7.6	15 (1.8)	1.0–2.9									
Auto-immune diseases	1 (1.1)	0.0–5.9	3 (0.4)	0.1–1.0									
Gestational comorbidities	9 (9.8)	4.6–17.8	71 (8.5)	6.7–10.6	1.2	0.5–2.5	0.6949	1.2	0.6–2.6	0.592			
Multiple pregnancy	2 (2.2)	0.2–7.6	22 (2.6)	1.7–4.0									
Other	8 (8.7)	3.8–16.4	54 (6.5)	4.9–8.4									
Hypertensive disorders	7 (7.6)	3.1–15.1	19 (2.3)	1.4–3.5	**3.5**	**1.2–9.1**	**0.0103**	**2.7**	**1.0–7.0**	**0.044**	**2.7**	**1.0–7.1**	**0.042**
Pre-gestational	4 (4.3)	1.2–10.8	15 (1.8)	1.0–2.9									
Gestational /Pre-eclampsia	4 (4.3)	1.2–10.8	6 (0.7)	0.3–1.6									
Diabetes	12 (13.0)	6.9–21.7	45 (5.4)	4.0–7.2	**2.6**	**1.2–5.3**	**0.0094**	**2.2**	**1.1–4.5**	**0.036**	**2.2**	**1.1–4.5**	**0.034**
Pregestational	4 (4.3)	1.2–10.8	8 (1.0)	0.4–1.9									
Gestational	8 (8.7)	3.8–16.4	37 (4.4)	3.1–6.1									
Unknown	0 (0.0)	n.a.	2 (0.2)	0.0–0.9									
**Maternal BMI**													
BMI > 30	28 (30.4)	21.3–40.9	180 (21.6)	18.8–24.5	**1.7**	**1.1–2.9**	**0.0220**	1.3	0.8–2.2	0.351	1.4	0.8–2.4	0.201
BMI > 35	15 (16.3)	9.4–25.5	66 (7.9)	6.2–10.0									
Unknown	12 (13.0)	6.9–21.7	110 (13.2)	11.0–15.7									
**COVID-19 exposure**													
Timing of exposure													
> 20 weeks gestation	84 (91.3)	83.6–96.2	742 (89.0)	86.6–91.0	1.1	0.5–2.8	0.8538	1.4	0.7–3.2	0.356			
Unknown	0 (0.0)	n.a.	11 (1.3)	0.7–2.3									

The effect of maternal characteristics known to be risk factors^7,8,17^ were tested (i.e. maternal age > 35 year old, obesity defined as a BMI > 30, hypertensive disorders (including pre-eclampsia), pre-and gestational diabetes, pre-existent pulmonary, cardiovascular, renal, oncologic diseases and immunosuppression), as well as pregnancy related risk factors such as pregnancy conditions (threatened preterm labor, placenta previa, placental malfunction and preterm premature rupture of the membrane (PPROM) (dichotomized as yes/no))), nulliparity (dichotomized as yes/no), ethnicity (defined as Caucasian yes/no), multiple pregnancy, age of pregnancy at infection (dichotomized as < or > 20 WG)^9^.

In bold are presented significant results.

SARS-CoV-2, Severe acute respiratory syndrome coronavirus 2; OR, odds ratio; aOR, adjuster odds ratio; y.o.; years old; BMI, Body Mass Index; n.a., non-applicable.

^a^ORs were calculated without missing values.

^b^Adjusted for specific COVID-19 risk factors (maternal age, pulmonary comorbidities, hypertensive disorders, diabetes mellitus, maternal BMI and other maternal comorbidities with a low prevalence in the cohort), specific pregnancy risk factors (ethnicity, parity, other pregnancy conditions (placenta previa, preterm premature rupture of the membrane , preterm labor, IUGR ) and timing of exposure.

^c^Adjusted for specific COVID-19 risk factors only (maternal age, pulmonary comorbidities, hypertensive disorders, diabetes mellitus, maternal BMI and other maternal comorbidities with a low prevalence in the cohort).

### Secondary outcomes

#### Absolute risk of pregnancy, obstetrical and neonatal outcomes

No differences were observed in terms of livebirth rate among positive women with severe adverse outcomes (i.e. cases) compared to women with no or mild adverse outcomes (i.e. controls) [absolute rate 92.6% (n = 75/81) compared to 98.1% (n = 656/669)] (Table 4), although a trend toward poorer obstetrical outcomes was observed among women with severe adverse outcomes [absolute rate of fetal loss > 14 WG 7.4% (n = 6/81) compared to 1.9% (n = 13/669)]. An increased risk of caesarean section was observed among patients with severe adverse outcomes [absolute caesarean sections rate 70.7% (n = 53/75) compared to 30.9% (n = 203/656)]. Similarly, women with severe maternal outcomes were at increased risk of preterm delivery < 37WG [absolute risk 62.7% (n = 32/51) compared to 36.3% (78/215)] and < 34 WG [absolute risk 51.9% (n = 14/27) compared to 20.5% (24/117)], most of which were iatrogenic [81.3% (n = 26/32) and 85.7% (n = 12/14), respectively]. Newborns born to mothers with severe adverse pregnancy outcomes were more frequently admitted to NICU [absolute risk 41.3% (n = 31/75) compared to 11.6% (n = 76/656)]. The most frequent reasons for admission were prematurity [71.0% (n = 22/31)] and respiratory distress [48.5% (n = 15/31)] (Table 4). A positive SARS-CoV-2 test at birth was observed in 2.9% of neonates (n = 11/384).) The rates of suspected perinatal transmission and reduced birthweight were similar between newborns born to mothers with severe outcomes compared to those with no or mild outcomes.

**Table 4:** Obstetrical and neonatal outcomes depending on maternal severity among women with a positive SARS-CoV-2 test.

Obstetrical/neonatal outcomes	Pregnant women with a positive test result for SARS-CoV-2			
	Severe adverse maternal outcomes n = 81		No/mild adverse maternal outcomes n = 669	
	n (%)	95% CI	n (%)	95% CI
**Pregnancy outcomes > 14 WG**				
Livebirth	75 (92.6)	84.6–97.2	656 (98.1)	96.7–99.0
Fetal loss > 14 WG	6 (7.4)	2.8–15.4	13 (1.9)	1.0–3.3
Termination of pregnancy	1 (1.2)	0.0–6.7	2 (0.3)	0.0–1.1
Obstetrical outcomes among livebirth	75		656	
GA at delivery (Weeks gestation)				
Median GA (IQR)	37 (34–38)		39 (38–40)	
Unknown GA at delivery	0 (0.0)	n.a.	2 (25.8)	15.1–41.0
Obstetrical management				
All vaginal deliveries	22 (29.3)	19.4–41.0	447 (68.1)	64.4–71.7
Vaginal delivery after spontaneous onset of labour	10 (45.5)	24.4–67.8	280 (62.6)	58.0–67.1
Vaginal delivery after induction of labour	12 (54.5)	32.2–75.6	167 (37.4)	32.9–42.0
Caesarean sections—no (%)	53 (70.7)	59.0–80.6	203 (30.9)	27.4–34.6
Elective caesarean sections—no (%)	21 (39.6)	26.5–54.0	85 (41.9)	35.0–49.0
Emergency pre-labor caesarean sections—no (%)	12 (22.6)	12.3–36.2	16 (7.9)	4.6–12.5
In labour caesarean sections after induction	12 (22.6)	12.3–36.2	52 (25.6)	19.8–32.2
In labour caesarean sections after spontaneous	8 (15.1)	6.7–27.6	50 (24.6)	18.9–31.2
Unknown	0 (0.0)	n.a.	6 (0.9)	0.3–2.0
Preterm birth among pregnancy with exposure < 37 WG	51		215	
All preterm birth < 37 WG—no (%)	32 (62.7)	48.1–75.9	78 (36.3)	29.8–43.1
Latrogenic birth among preterm birth—no (%)	26 (81.3)	63.6–92.8	49 (62.8)	51.1–73.5
Unknown—no (%)	0 (0.0)	n.a.	1 (1.3)	0.0–6.9
Unknown GA at delivery	0 (0.0)	n.a.	1 (0.5)	0.1–2.6
Preterm birth among pregnancy with exposure < 34WG	27		117	
All preterm birth < 34 WG—no (%)	14 (51.9)	31.9–71.3	24 (20.5)	13.6–29.0
Latrogenic birth among preterm birth—no (%)	12 (85.7)	57.2–98.2	14 (58.3)	36.6–77.9
Unknown—no (%)	0 (0.0)	n.a.	0 (0.0)	n.a.
Unknown GA at delivery	0 (0.0)	n.a.	1 (0.9)	0.0–4.7
Neonatal outcomes among livebirths	75		656	
Neonatal death	0 (0.0)	n.a.	1 (0.2)	0.0–0.8
NICU admission—no (%)				
All NICU admission	31 (41.3)	30.1–53.4	76 (11.6)	9.2–14.3
Prematurity	22 (71.0)	52.0–85.8	32 (42.1)	30.9–54.0
Respiratory distress	15 (48.4)	30.2–66.9	18 (23.7)	14.7–34.8
Sepsis	0 (0.0)	n.a.	5 (6.6)	2.2–14.7
Cardiovascular complications	0 (0.0)	n.a.	0 (0.0)	n.a.
Hypoglycemia	0 (0.0)	n.a.	10 (13.2)	6.5–22.9
Hyperbilirubinemia	1 (3.2)	0.1–16.7	9 (11.8)	5.6–21.3
Coagulopathy	0 (0.0)	n.a.	0 (0.0)	n.a.
Neurologic complications	0 (0.0)	n.a.	2 (2.6)	0.3–9.2
Other	3 (9.7)	2.0–25.8	19 (25.0)	15.7–36.3
Unknown	5 (6.7)	2.2–14.9	47 (7.2)	5.3–9.4
SARS-CoV-2 perinatal transmission rates				
Total of SARS-CoV-2 test at birth—no (%)	44 (58.7)	46.7–69.9	340 (51.8)	47.8–55.7
Suspected SARS CoV-2 perinatal transmission (positive RT-PCR at birth)—no (%)	2 (4.5)	0.6–15.5	9 (2.6)	1.2–5.0
Birthweight				
Birthweight < P10—no (%)	1 (1.3)	0.0–7.2	39 (5.9)	4.3–8.0
Unknown	5 (6.7)	2.2–14.9	12 (1.8)	0.9–3.2

Obstetrical and neonatal outcomes among positive women were assessed based on the severity of maternal disease through a case control study comparing positive women with severe adverse maternal outcomes (cases) to positive women with no or mild adverse maternal outcomes (control).

SARS-CoV-2, severe acute respiratory syndrome coronavirus 2; CI, confidence interval; WG, weeks ‘gestation; GA, gestational age; NICU, Neonatal Intensive Care Unit; n.a., non-applicable.

## Discussion

In this study, we present the largest cohort of pregnant women tested for SARS-Cov-2 worldwide and the first analysis of primary data stratified by the severity of maternal disease, allowing us to identify specific risk factors associated with adverse maternal outcomes.

Severe adverse outcomes, defined by maternal death, admission to ICU and/or advanced oxygen support were observed in 9.9% of cases. Pulmonary comorbidities, hypertensive disorders and diabetes mellitus were significantly associated with an increased risk of severe maternal outcomes, while usual pregnancy related risk factors were not. No difference in the livebirth rate was observed between pregnant women with severe adverse outcomes and patients with an uncomplicated course. Nevertheless, a significant increased risk of caesarean section, preterm birth and neonatal admission to the intensive care unit was observed, highlighting that obstetrical and neonatal outcomes are influenced by the severity of maternal disease.

The rate of severe disease observed here is similar to what has been previously reported in other large cohorts^3–5^ and summarized in a recent meta-analysis^6^,where the risk of severe disease among pregnant women with COVID-19 was estimated to be 13% (95%CI 6–21%). Importantly, this risk of severe maternal complications appears significantly higher when compared to a non-pregnant population at an equivalent age, with an increased odds of ICU admission or mechanical ventilation up to 1.6 (95%CI 1.3–2.0) and 1.9 (95%CI 1.4–2.6) respectively^6^.

Risk factors for severe maternal disease appear to be similar to what has been previously described in the general population, namely pulmonary pathologies, hypertension and diabetes^7,8^. Congruently, in their meta-analysis, Allotey et al. observed an increased risk of severe disease among pregnant women > 35 y.o., those with chronic hypertension, pre-existing diabetes, or body mass index > 30^6^. Interestingly, in our study, after adjustment, obesity was not independently associated with an increased risk of severe adverse outcomes. This could be explained by the fact that overweight patients often suffer from hypertension and diabetes (metabolic syndrome), which could act as the predominant causal factors. Both are associated with macro- and micro-vascular complications, and endothelial dysfunction has been suggested as a major pathophysiological mechanism associated with COVID-19 severity^18,19^. In pregnancy, endothelial change is a well-known mechanism of obstetrical complications, such as gestational hypertension, HELLP (Hemolysis, elevated liver enzymes, low platelets) and pre-eclampsia^20^, and may contribute to the increased risk of COVID-19 complications. In our study, we did not observe any association with maternal age. This could be explained by the low number of patients > 35 y.o. included. Similarly, ethnicity (non-Caucasian versus Caucasian) was not associated with poorer outcomes, unlike previously described^21^.

We observed a 2.9% rate of positive test among newborns born to mothers with a positive SARS-CoV-2 test. The clinical relevance of this finding remains unclear, as, at the time of the study, we were lacking comprehensive data regarding COVID-related symptoms or COVID-suspected symptoms among newborns, repeated testing and long-term follow-up. Perinatal transmission of SARS-CoV-2 has been reported by others, both in case of vaginal and cesarean sections, and was associated in some cases with neonatal symptoms^1,4,22^. In all reported cases, the possibility of postnatal infection through contacts with parents or medical personal remains difficult to exclude^1,4^. Alternatively, transplacental transmission has been suspected in few cases, where specific IgM were detected among newborns^23,24^. Nevertheless, perinatal/vertical transmission appear to be rare and mainly associated with good neonatal outcomes^1,4,23,24^.

Our study has several limitations. First, we present here the outcomes among pregnant women with a confirmed SARS-CoV-2 infection and therefore only observational conclusions can be drawn regarding the absolute risks of severe disease and adverse obstetrical/neonatal outcomes, as a control group of negative patients was not included. Nevertheless, this was beyond the scope of the present study, whose first aim was to identify specific risk factors.

Second heterogeneities exist between participating centers in the testing of pregnant women. While some centers performed routine systematic screening of presenting women independently of compatible symptoms, other only tested symptomatic pregnant women. This could have led to a selections bias of more severe symptomatic COVID-19 cases. If a symptomatic SARS-CoV-2 infection is associated with poorer maternal, obstetrical and neonatal outcomes, this selection bias may have resulted in an overestimation of the absolute risk of adverse outcomes. However, the rate of asymptomatic infections among included positive women of 31.9% (n = 295/926) is quite similar to the rate of asymptomatic infection described in the general population, estimated to range around 40–45%^25,26^ and suggests a low impact of this potential bias. Similarly, patients admitted with severe disease were very likely systematically tested for SARS-CoV-2, which may have led to a possible overestimation of the actual rate of severe adverse outcome among positive patients. Follow-up analysis, including patients with ongoing pregnancies with an uncomplicated course based on systematic screening will help assess the exact risk in a more general population of pregnant women.

Third, most patients were included during the 3rd trimester of gestation, with the majority included close to delivery, while 130 pregnancies were still ongoing at the time of analysis. Although, we did not observe any impact of the gestational age (i.e. > 20 WG) on the severity of maternal disease, this could be related to a lack of statistical power. Pregnancy-related vascular complications only occur after 20 WG, which would suggest an increased risk of maternal complications in cases of maternal infection at a later stage of the pregnancy, as observed by others^9^. In our cohort, severe maternal outcomes were also observed in women exposed at < 20 WG, with an overall similar risk (n = 8/89, 9.0%) to what was described in the whole cohort. Therefore, caution should also be taken with pregnant women infected in early pregnancy.

Although our data regarding obstetrical outcomes are reassuring, definite conclusions cannot be drawn. Infections occurring at an earlier stage of gestation may be associated with poorer obstetrical outcomes. Viral particles have been detected within the placentas of women infected earlier during pregnancy^10,12,13,27^. Although placental infection seems rare, it has been associated with evidence of malperfusion^28–30^, which is known to be associated with reduced fetal growth and intra-uterine fetal death. Of note, Khalil et al. have shown an increase in the number of stillbirths during the epidemic peak, without being able to determine whether this is a direct effect of the virus^31^. At the time of analysis, pregnancies < 37WG that were exposed during the 1st and 2nd-trimesters were still ongoing (Fig. 1), suggesting an uncomplicated course. Subsequent analysis, including those patients, are needed to better define obstetrical and neonatal outcomes.

In conclusion, pregnant women, particularly those with associated comorbidities, seem to be at higher risk of severe complications of SARS-CoV-2 infection. Obstetrical and neonatal outcomes appear to be influenced by the severity of maternal disease; complications include caesarean sections, neonatal prematurity and neonatal admission to the intensive care unit. Further studies are needed to assess maternal and neonatal outcomes for cases of earlier exposure.

## Supplementary Information

Supplementary Information.
