# Supplementary material for: Maternal outcomes and risk factors for COVID-19 severity among pregnant women
Source: Sci Rep. 2021 Jul 6;11:13898. doi: 10.1038/s41598-021-92357-y (PMC8260739; doi:10.1038/s41598-021-92357-y)
Supplement: Supplementary file 1 — Supplementary Information. [file 41598_2021_92357_MOESM1_ESM.docx]

**SUPPLEMENTARY MATERIAL:**

**MATERNAL OUTCOMES AND RISK FACTORS FOR COVID-19 SEVERITY AMONG PREGNANT WOMEN**

Manon Vouga^*1^, Guillaume Favre*^1^, Oscar Martinez-Perez*^2^, Leo Pomar*^1,^ Laura Forcen Acebal^3^, Alejandra Abascal-Saiz^4^, Maria Rosa Vila Hernandez^5^, Najeh Hcini^6^, Véronique Lambert^6^, Gabriel Carles^6^, Joanna Sichitiu^1, 7^, Laurent Salomon^7^, Julien Stirnemann^7^, Yves Ville^7^, Begoña Martinez de Tejada^8^, Anna Goncé^9^, Ameth Hawkins-Villarreal^9^, Karen Castillo^9^, Eduard Gratacos Solsona^9^, Lucas Trigo^10^, Brian Cleary^11^, Michael Geary^12^, Helena Bartels^12^, Feras Al-Kharouf^12^, Fergal Malone^12^, Mary Higgins^13^, Niamh Keating^14^, Susan Knowles^15^, Christophe Poncelet^16^, Carolina Carvalho Ribeiro-do-Valle^17^, Fernanda Garanhani Surita^17^, Amanda Dantas-Silva^17^, Carolina Borrelli^17^, Adriana Gomes Luz^17^, Javiera Fuenzalida^18^, Jorge Carvajal^18^, Manuel Guerra Canales^19^, Olivia Hernandez^20^, Olga Grechukhina^21^, Albert I. Ko^22^, Uma Reddy^22^, Rita Figueiredo^23^, Marina Moucho^23^, Pedro Viana Pinto^23^, Carmen De Luca^24^, Marco De Santis^24^, Diogo Ayres de Campos^25^, Inês Martins^25^, Charles Garabedian^26^, Damien Subtil^26^, Betania Bohrer^27^, Maria Lucia Da Rocha Oppermann^28^, Maria Celeste Osorio Wender^28^, Lavinia Schuler-Faccini^29^, Maria Teresa Vieira Sanseverino^29^, Camila Giugliani^30^, Luciana Friedrich^27^, Mariana Horn Scherer^29^, Nicolas Mottet^31^, Guillaume Ducarme^32^, Helene Pelerin^33^, Chloe Moreau^33^, Bénédicte Breton^34^, Thibaud Quibel^35^, Patrick Rozenberg^35^, Eric Giannoni^1^, Cristina Granado^36^, Cécile Monod^36^, Doris Mueller^36^, Irene Hoesli^36^, Dirk Bassler^37^, Sandra Heldstab^38^, Nicole Ochsenbein Kölble^39^, Loïc Sentilhes^40^, Melissa Charvet^40^, Jan Deprest^41^, Jute Richter^41^, Lennart Van der Veeken^42^, Béatrice Eggel-Hort^43^, Gaetan Plantefeve^44^, Mohamed Derouich^45^, Albaro José Nieto Calvache^46^, Maria Camila Lopez-Giron^46^, Juan Manuel Burgos-Luna^46^, Maria Fernanda Escobar-Vidarte^46^, Kurt Hecher^47^, Ann-Christin Tallarek^47^, Eran Hadar^48^, Karina Krajden Haratz^49^, Uri Amikam^50^, Gustavo Malinger^50^, Ron Maymon^51^, Yariv Yogev^49^, Leonhard Schäffer^52^, Arnaud Toussaint^53^, Marie-Claude Rossier^54^, Renato Augusto Moreira De Sa^55^, Claudia Grawe^56^, Karoline Aebi-Popp^57^, Anda-Petronela Radan^58^, Luigi Raio^58^, Daniel Surbek^58^, Paul Böckenhoff^59^, Brigitte Strizek^59^, Martin Kaufmann^60^, Andrea Bloch^61^, Michel Boulvain^62^, Silke Johann^63^, Sandra Andrea Heldstab^64^, Monya Todesco Bernasconi^64^, Gaston Grant^65^, Anis Feki^65^, Anne-Claude Muller Brochut^66^, Marylene Giral^67^, Lucie Sedille^67^, Andrea Papadia^68^, Romina Capoccia Brugger^69^, Brigitte Weber^70^, Tina Fischer^71^, Christian Kahlert^72^, Karin Nielsen Saines^73^, Mary Cambou^74^, Panagiotis Kanellos^75^, Xiang Chen^76^, Mingzhu Yin^77^, Annina Haessig ^78,^ Sandrine Ackermann^1^, David Baud*^1^, Alice Panchaud*^79,80^

^1^ Materno-fetal and Obstetrics Research Unit, Department “Femme-Mère-Enfant”, University Hospital, Lausanne, Switzerland; ^2^Obstetricia y Ginecologia, Puerta de Hierro University Hospital, Madrid, Spain ; ^3^Obstetrics and Gynaecology Department, 12 de Octubre University Hospital, Madrid, Spain; ^4^La Paz Universitary Hospital, Madrid, Spain ; ^5^ Hospital Santa Caterina, Girona, Spain; ^6^Department of Obstetrics and Gynaecology, CHOG, Saint-Laurent du Maroni, France; ^7^Obstétrique et de Médecine fœtale, Hopital Necker-Enfants malades, Paris, France; ^8^Obstetrics Division, Department of Pediatrics Gynecology and Obstetrics, University Hospitals of Geneva, Geneva, Switzerland; ^9^BCNatal Maternal-Fetal Medicine Service, Hospital Clínic, University of Barcelona, Barcelona, Spain; ^10^Fetal Medicine Research Center, Hospital Clínic and Hospital Sant Joan de Déu, Barcelona, Spain; ^11^Service of Pharmacy, The Rotunda Hospital, Dublin, Ireland;^12^Maternal-fetal medicine, The Rotunda Hospital, Dublin, Ireland; ^13^UCD Perinatal Research Centre, National Maternity Hospital, Dublin, Ireland; ^14^Maternal Medicine, National Maternity Hospital, Dublin, Ireland; ^15^Microbiology, National Maternity Hospital, Dublin, Ireland; ^16^ Obstetric and Gynecology unit, Centre Hospitalier René Dubos, Cergy-Pontoise, France;^17^ Department of Obstetrics & Gynecology, University of Campinas, Campinas, Brazil ; ^18^ Maternal-Fetal Medicine, Department of Obstetrics, Escuela de Medicina, Pontificia Universidad Católica de Chile, Santiago, Chile ; ^19^ Medicina Materno Fetal, Hospital San José, Santiago, Chile ; ^20^ Hospital Felix Bulnes Cerda Santiago Chile ; ^21^ Department of Obstetrics, Gynecology and Reproductive Sciences, Yale School of Medicine, New Haven, Connecticut, United States of America ; ^22^ Department of Epidemiology of Microbial Diseases, Yale of School of Public Health, New Haven, Connecticut, United States of America ; ^23^ Serviço ginecologia e obstetrícia, Centro Hospitalar e universitário São João, Porto, Portugal ; ^24^ Teratology Information Service, Fondazione Policlinico Universitario Agostino Gemelli IRCCS, Roma, Italia ; ^25^ Medical School, Santa Maria University Hospital, Lisbon, Portugal ; ^26^ Department of Obstetrics, Jeanne de Flandre University Hospital, Lille, France ; ^27^ Pediatra e Neonatologista, Hospital de Clinicas de Porto Alegre, Universidade Federal do Rio Grande do Sul, Porto Alegre, Brazil ; ^28^ Maternity Ward, Hospital de Clinicas de Porto Alegre, Universidade Federal do Rio Grande do Sul, Porto Alegre, Brazil ; ^29^ Departamento de Genética, Hospital de Clinicas de Porto Alegre, Universidade Federal do Rio Grande do Sul, Porto Alegre, Brazil ; ^30^ Hospital de Clinicas de Porto Alegre, Universidade Federal do Rio Grande do Sul Porto Alegre Brazil ; ^31^ Department of Obstetrics and Gynecology, Université de Franche Comté, Besançon, France ; ^32^ Departement of Obstetrics and Gynecology, Centre Hospitalier Departemental de Vendée, La Roche sur Yon, France ; ^33^ Clinical Research Department, Centre Hospitalier Départemental de Vendée, La Roche sur Yon, France ; ^34^Department of Obstetrics and Gynecology, Annecy Genevois Hospital, Annecy, France ; ^35^Department of Gynecology and Obstetrics, Intercommunal Hospital Centre of Poissy-Saint-Germain-en-Laye, Poissy, France ; ^36^ Department of Obstetrics and Antenatal Care, University Hospital Basel, Basel, Switzerland ; ^37^ Department of Neonatology, UniversitätsSpital Zürich, Zurich, Switzerland ; ^38^ Department of Anthropology, University of Zurich, Zurich, Switzerland ; ^39^ Clinic of Obstetrics, UniversitätsSpital Zürich, Zurich, Switzerland ; ^40^ Department of Obstetrics and Gynecology,Bordeaux University Hospital, Bordeaux, France ; ^41^ Department of Obstetrics and Gynecology, University Hospitals Leuven, Leuven, Belgium ; ^42^ Department of Regeneration and Development, Katholieke Universiteit Leuven, Leuven, Belgium ; ^43^ Obstetric and gynecology unit, Sion hospital, Sion Switzerland ; ^44^ Service de Réanimation polyvalente et USC, Victor Dupouy Hospital, Argenteuil, France ; ^45^ Obstetrics unit, Victor Dupouy Hospital, Argenteuil, France ; ^46^ Obstetrics and gynecology department, Fundacion Clinica Valle de Lili, Universitary Hospital, Cali, Colombia ; ^47^Department of Obstetrics and Fetal Medicine, University Medical Center Hamburg-Eppendorf, Hamburg, Germany ; ^48^ Maternal-Fetal Medicine Unit, Rabin Medical Center, Tel-Aviv University, Tel Aviv, Israel ; ^49^ Division of Ultrasound in Obstetrics and Gynecology, Lis Maternity Hospital, Tel Aviv, Israel ; ^50^ Division of Ultrasound in ObGy, Tel Aviv Sourasky Medical Center, Tel Aviv, Israel ; ^51^ Israeli Society of Obstetrics and Gynecology, Hasaf Harofe Medical Center, Tel Aviv University, Tel Aviv, Israel ; ^52^Obstetrics Cantonal Hospital of Baden, Affiliated Hospital of the University of Zurich, Baden, Switzerland ; ^53^ Department of Gynecology and Obstetrics, Intercantonal Hospital of Broye, Payerne, Switzerland ; ^54^ Obstetrics and Gynecology, Hospital Riviera Chablais, Rennaz, Switzerland ; ^55^ Maternal-Fetal Unit, Federal Fluminense University, Rio de Janeiro, Brazil ; ^56^ Department of Obstetrics, Gynecology Stadtspital Triemli Zürich Zurich Switzerland ; ^57^ Department of Infectious Diseases, University Hospital Bern, Bern, Switzerland ; ^58^ Department of Obstetrics and Gynecology, Inselspital, Bern, Switzerland ; ^59^ Department of Obstetrics and Prenatal Medicine, University Hospital Bonn, Bonn, Germany ; ^60^ Obstetric and Gynecology Unit, Spital Bülach,Bülach, Switzerland ; ^61^ Obstetric and Gynecology Unit, Hopital du Jura, Delémont, Switzerland ; ^62^ Pôle Department of Gynecology and obstetrics, GHOL Hôpital de Nyon, Nyon, Switzerland ; ^63^ Department of Obstetrics and Gynecology Spitalzentrum OBerwallis, Standort Visp, Visp, Switzerland ; ^64^ Frauenklinik, Kantonsspital Aarau, Aarau, Switzerland ; ^65^ Department of Obstetrics and Gynecology, HFR Fribourg hospital, Fribourg, Switzerland ; ^66^ GynEcho medical practice, Fribourg, Switzerland ; ^67^ Department of Obstetrics and Gynecology , La Rochelle hospital, La Rochelle, France ; ^68^Department of Obstetrics and Gynecology, Ente Ospedaliero Cantonale of Lugano, Lugano, Switzerland ; ^69^ Department of Obstetrics and Gynecology Réseau Hospitalier Neuchâtelois, Neuchâtel, Switzerland ; ^70^ Department of Obstetrics and Gynecology, Kantonsspital Obwalden (KSOW), Sarnen, Switzerland ; ^71^ Frauenklinik, Kantonsspital Saint Gall, Saint Gall, Switzerland ; ^72^Infectious Diseases and Hospital Epidemiology, Kantonsspital Saint Gall, Saint Gall, Switzerland ; ^73^ Department of Pediatrics, Division of Infectious Diseases, David Geffen UCLA School of Medicine, Los Angeles, CA, United States of America ; ^74^ Cardiac Surgery Department, David Geffen UCLA School of medicine, Los Angeles, CA, United States of America ; ^75^ Department of Obstetrics and Gynecology, Kantonsspital Uri, Altdorf, Switzerland ; ^76^ Dermatology unit, Xiangya Hospital, Changsha, China ; ^77^ Hunan Engineering Research Center of Gynecology and Obstetrics Disease, Xiangya Hospital, Changsha, China ; ^78^ Department of Obstetrics and Gynecology, Zuger Kantonsspital, Zug, Switzerland; ^79^Institute of Primary Health Care (BIHAM), University of Bern, Switzerland; ^80^Service of Pharmacy, Lausanne University Hospital and University of Lausanne, Switzerland.

* equally contributed to the work

**Corresponding author:** Prof. David Baud, MD PhD

Materno-fetal & Obstetrics Research Unit

Department of Obstetrics and Gynecology

Centre Hospitalier Universitaire Vaudois (CHUV)

1011 Lausanne – SWITZERLAND

Phone: (00) 41 79 556 13 51

Email: [david.baud@chuv.ch](mailto:david.baud@chuv.ch)

**Annex 1: COVI-PREG REGISTRY: QUALITY CHECKS**

COVI-Preg is an international registry build on the expertise acquired through the international registry for Zika virus in pregnancy. While developing the registry, several strategies were undertaken to reduce data errors and perform quality checks. This document describes these strategies.

**Data collection approach**

Data were collected using electronic case report forms (eCRFs) provided by a secured web-based survey build with REDCap (Research Electronic Data Capture),(Harris et al. 2009; 2019) a web application for building and managing online surveys and databases (projectredcap.org). This web applications allows a data quality check already during data entry by implementing:

1. Required items: variables considered important for the data reliability or necessary to define the exposure, as well as important confounding variables and outcomes by the scientific committee e.g. Date of oral information, SARS-CoV 2 SCREENING: pharyngeal and/or deep nasopharyngeal swab and RT-PCR, other virus screening, pregnancy outcomes). For these variables, error signs appear when data are missing.
2. Conditions: built-in restrictions which allow specific questions only to pop-up based on answers to a prior question. This user-friendly case report form environment using branching logic to reduce fields to fill in ensures that the person entering the data takes the right step, avoids errors and saves valuable time. (e.g. by choosing “Outpatient management” at the section “COVID-19 patient management” the section asking for inpatient management will not open).
3. Validations: provide direct feedback on the content of the filled fields. When an impossible or unlikely value is entered, a warning pops up. Data validation rules incorporated included predefined range of values, logical checks or error system to avoid incompatible or implausible answers (e.g. Systolic blood pressure less than 300mmHg, Error system by ticking “livebirth” as pregnancy outcome and entering a gestational age at delivery less than 23 weeks of gestation).
4. Help texts: ensure uniform data collection across all hospitals. Help texts with unambiguous definitions were added where necessary. Details and units for each question with a risk of misunderstanding were added. Conversion rules were also indicated (i.e.: Creatininemia (μmol/L) with 1 mg/dL = 88 μmol/L).
5. Limited number of open-ended questions: the questions were developed to avoid the maximum of free text section giving priority to “Yes/No” questions or multiple choices questions.
6. A signaling list of patients with missing information or incorrect data: All notifications of inconsistent information are collected on a patient level and bundled on a list for all patients registered by participating center allowing a dynamic data quality check so errors can be rectified quickly and easily.

A pilot testing was performed using the data collection platform by the research team in Lausanne using 10 virtual patient cases that were entered by three different persons to test the fluidity of the eCRFs and the consistence of the answers provided. In a second phase, the first patients included locally were used to continue the assessment and improvement of the eCRFs.

The eCRF are easily adaptable at any time by adding or editing queries online (e.g. serological testing was added once available and informed participating centers per email of this new item).

**Data quality requirements and internal quality control at participating centers**

Collaborators at participating centers were registered in our platform, identified by data access group identification, name, e-mail address, and institution name. Each center had access to its own data only. One person per site was assigned the task of data collection, correction of systematic inconsistencies and aberrant value checks and implementation of internal quality control procedures in place, with a second person at the site double checking data quality and entry against source documents. Participating centers received a document detailing the procedures, which described the access to the data collection platform. Video tutorials were also created demonstrating how to use the REDCap system adapted to the COVI-PREG platform, at <http://video.en.covi-preg.ch>. Site investigators also received a data dictionary detailing every information to be gathered with a review of potential answer.

All sites had Institutional Review Board (IRB) approval to participate in the COVI-Preg study and were not allowed to initiate any study procedures before our central data unit received documentation of IRB approval.

**External data quality check at the coordinating center**

Lausanne University hospital, Switzerland, is the lead coordinating center for the COVI-PREG registry. A dedicated team composed by a registry coordinator, obstetricians, an epidemiologist, a legal adviser and an IT adviser worked full time to carry out the project, monitor the data and assist collaborators in every step of our collaborative study with the registry from site enrollment to data collection and data checks.

Regular conference calls with participating centers were organized to discuss recruitment issues, data problems or any other questions or comments regarding the registry. Inquiries were also enabled by e-mail to ensure easy access to all registry participants and to optimize registry quality.

No external monitoring by the coordinating center was performed as travel restrictions linked to the COVID-19 pandemic prevented external monitoring by direct auditing of source documents. Furthermore, such external quality check would have made the study unfeasible as the study has more 200 participating across the world. The registry was a voluntary international collaborative effort and funding was not available for contracting local auditing companies to perform medical record review. This requirement would have made the study untenable. Nevertheless, the study staff in Lausanne regularly checked data using statistical tests, and manual table controls for any discrepancies. When an error was suspected, the lead collaborator of the participating center and/or the data entry team member was asked to double check the information with queries sent to the participating site requesting review and correction of any information that was found to be be inaccurate. Random individual data examinations were also performed through the REDCap system. Finally, short interim analyses using the REDCap system were carried out to monitor the global relevance of the data and results were compared to other analyses available in the scientific literature. Our findings were comparable to those of prior smaller studies.

**Data source**

All registry data is available to the journal for review. Local site investigators can provide verification of de-identified source documentation to the data center upon request.

**STATISTICAL ANALYSIS**

**Missing values.** Based on the assumption that severe conditions are typically well documented in a registry, maternal comorbidities and pregnancy conditions were considered as negative if not reported. Based on the hypothesis of missing variables completely at random (MCAR), multiple imputations were performed to increase the power of comparisons and estimate the risks while taking into account missing data on maternal body mass index (BMI), parity, timing of exposure and maternal origin.

**TABLE S1 – COVI-Preg participating centers**

|  |  |  |  |
| --- | --- | --- | --- |
|  |  |  |  |
|  |  |  | Nb of cases included |
| **Countries** | |  | in the registry |
|  |  |  | as per July 26th |
|  |  |  |  |
|  |  |  |  |
| **Belgium** | |  | **8** |
|  | UZ Leuven, Leuven |  | 8 |
| **Brazil** | |  | **51** |
|  | Unicamp, Sao Polo |  | 34 |
|  | Universidad Federal do Rio Grande do Sul (UFRGS), Porto Alegre |  | 13 |
|  | National Institute of Women, Children and Adolescents Health, Fernandes Figueira (IFF), Rio de Janeiro |  | 4 |
| **Canada** | |  | **2** |
|  | Ottawa Hospital, Ottawa |  | 2 |
| **Chile** | |  | **29** |
|  | San José hospital, San José |  | 29 |
| **Colombia** | |  | **6** |
|  | Valle de Lili Foundation university hospital, Cali |  | 6 |
| **France** | |  | **144** |
|  | Hôpital Necker, Paris |  | 53 |
|  | Groupement hospitalier Atlantique , La Rochelle |  | 2 |
|  | Centre Hospitalier Annecy Genevois, Annecy |  | 1 |
|  | Lilles University Hospital, Lille |  | 13 |
|  | Centre Hospitalier René-Dubos, Pontoise |  | 25 |
|  | Bordeaux University Hospital, Bordeaux |  | 9 |
|  | Centre Hospitalier Départemental Vendée, La Roche-sur-yon |  | 14 |
|  | Centre Hospitalier d'Argenteuil, Argenteuil |  | 6 |
|  | Besancon University Hospital, Besancon |  | 9 |
|  | Hôpital intercommunal Poissy-Saint-Germain-en-Laye, Poissy |  | 12 |
| **French Guyana** | |  | **74** |
|  | Centre Hospitalier de l'Ouest Guyannais, Saint-Laurent du Maroni |  | 74 |
| **Germany** | |  | **9** |
|  | Hamburg University Hospital, Hamburg |  | 6 |
|  | Bonn university hospital, Bonn |  | 3 |
| **Ireland** | |  | **43** |
|  | The National Maternity Hospital, Dublin |  | 19 |
|  | Rotunda Hospital, Dublin |  | 24 |
| **Italia** | |  | **12** |
|  | Policlinico Universitario Fondazione Agostino Gemelli, Roma |  | 12 |
| **Israel** | |  | **5** |
|  | Rabin Medical Center, Tel Aviv |  | 5 |
| **Portugal** | |  | **35** |
|  | Santa Maria university hospital, Lisbon |  | 12 |
|  | São João university hospital, Porto |  | 23 |
| **Spain** | |  | **522** |
|  | Barcelona University Hospital (CLINIC), Barcelona |  | 43 |
|  | Puerta de Hierro University Hospital, Madrid |  | 479 |
| **Switzerland** | |  | **113** |
|  | Lausanne University Hospital, Lausanne |  | 21 |
|  | Geneva University Hospitals, Geneva |  | 43 |
|  | Hôpitaux du Valais, Sion |  | 5 |
|  | Bern University Hospital (Insel), Bern |  | 3 |
|  | Jura Hospital, Delemont |  | 3 |
|  | Ente Ospedaliero Cantonale (EOC), Belinzone |  | 1 |
|  | Hôpital intercantonal de la Broye, Payerne |  | 4 |
|  | Obwald Cantonal Hospital |  | 1 |
|  | Triemli Hospital Zurich, Zurich |  | 4 |
|  | Baden Canton Hospital, Baden |  | 1 |
|  | Groupement hospitalier de l'Ouest lémanique, Nyon |  | 3 |
|  | Aarau cantonal hospital, Aarau |  | 2 |
|  | Basel University Hospital, Basel |  | 6 |
|  | Zurich University Hospital, Zurich |  | 10 |
|  | Saint Gallen Canton Hospital, Saint-Gallen |  | 1 |
|  | Hôpital Riviera-Chablais, Rennaz |  | 4 |
|  | Uri Kantonspital |  | 1 |
| **United states of America** | |  | **26** |
|  | Yale School of Medicine, New-York |  | 18 |
|  | University of California, Los Angeles |  | 8 |
|  |  |  |  |
|  | **Total of cases included:** |  | 1079 |
|  |  |  |  |
|  |  |  |  |
